# Supplementary material for: MetaRibo-Seq measures translation in microbiomes
Source: Nat Commun. 2020 Jun 29;11:3268. doi: 10.1038/s41467-020-17081-z (PMC7324362; doi:10.1038/s41467-020-17081-z)
Supplement: Supplementary file 10 — Supplementary Data 7 [file 41467_2020_17081_MOESM10_ESM.zip › File2/Confidence_High_Taxonomy/364074_out.krona.html]

Javascript must be enabled to view this page.

members
magnitude
magnitudeUnassigned
count
unassigned
taxon
rank

364074\_out

2

2
superkingdom
2

phylum
2
1239

186801
2
class

order
2
186802

family
2
541000

1263
2
genus

2
species

SRS077502\_contig\_number\_17827SRS1041091\_contig\_number\_20743
2293151
